# Serological Evidence and Coexposure of Selected Infections among Livestock Slaughtered at Eastern Cape Abattoirs in South Africa

**DOI:** 10.1155/2023/8906971

**Published:** 2023-12-01

**Authors:** K. D. Mazwi, F. B. Kolo, I. F. Jaja, R. P. Bokaba, Y. B. Ngoshe, A. Hassim, L. Neves, H. van Heerden

**Affiliations:** ^1^Department of Veterinary Tropical Diseases, Faculty of Veterinary Science, University of Pretoria, Onderstepoort, Pretoria, South Africa; ^2^Department of Livestock and Pasture Science, Faculty of Science and Agriculture, University of Fort Hare, Alice, South Africa; ^3^Department of Agriculture and Animal Health, University of South Africa, Roodepoort, Johannesburg, South Africa; ^4^Epidemiology Section, Department of Production Animal Studies, Faculty of Veterinary Science, University of Pretoria, Onderstepoort, Pretoria, South Africa; ^5^Centro de Biotecnologia, Universidade Eduardo Mondlane, Maputo, Mozambique

## Abstract

Zoonotic infections were investigated in a cross-sectional study on asymptomatic livestock slaughtered in abattoirs in the Eastern Cape. Antibodies against *Brucella* spp., *Coxiella burnetii*, *Toxoplasma gondii*, and the coexposure were investigated in sera using serological tests. A total of 565 animals comprising of 280 cattle, 200 sheep, and 85 pigs were screened using RBT, iELISA, CFT, and AMOS-PCR. The Mast® Toxoreagent test and iELISA were used for the detection of *T. gondii* and *C. burnetii*, respectively. The *Brucella* positivity based on at least two tests was 4.3% (12/280), 1.0% (2/200), and 0.0% (0/85) in cattle, sheep, and pigs, respectively. *Toxoplasma gondii* seropositivity of 37.90% (106/280), 1.50% (3/200), and 7.10% (6/85) was observed in cattle, sheep, and pigs, respectively. *Coxiella burnetii* seropositivity of 26.40% (74/280), 15.00% (30/200), and 2.40% (2/85) was observed in cattle, sheep, and pigs, respectively. Coexposure was detected in cattle for positivity against *C. burnetii* and *T. gondii* 40.54%, *Brucella* spp. and *T. gondii* 1.35%, and *Brucella* spp. and *C. burnetii* 4.05%. Coexposure for *Brucella* spp., *C. burnetii*, and *T. gondii* 4.05% was detected in cattle. Coexposure of *Brucella* spp. and *C. burnetii* 6.67% was detected in sheep. The AMOS-PCR identified *B. abortus* in cattle and a mixed infection of *B. abortus* and *B. melitensis* in sheep in 64.71% seropositive samples. To our knowledge, the coexposure of *Brucella* spp., *T. gondii*, and *C. burnetii* in cattle has not been reported. Coexposure of *Brucella* spp. and *C. burnetii* in cattle and sheep is significant as it results in reproductive losses and constitutes an infectious risk to humans. The detection of antibodies against multiple zoonotic infections in livestock from abattoirs has implications for public health.

## 1. Introduction

According to a study conducted by the Asian Pacific Strategy for Emerging Diseases in 2010, approximately 60% of emerging human diseases are zoonotic and more than 70% of these pathogens come from wildlife species [1]. It has been reported that over 14 million deaths are reported annually due to infectious diseases [2]. Zoonotic diseases affect human and animal populations, resulting in illnesses, mortality, and decreased productivity [3]. These diseases can have a significant social impact in endemic areas [3]. Many pathogens causing abortion in animals may lead to severe human illness, particularly *Toxoplasma gondii*, *Brucella* spp., *Chlamydophila* spp., *Campylobacter* spp., *Salmonella* spp., *Listeria* spp., *Leptospira* spp., and *Coxiella burnetii* [4, 5]. In South Africa (SA), brucellosis, Rift Valley fever, and toxoplasmosis have been identified as part of the seventeen diseases classified as zoonotic priorities [6]. There is currently a dearth of information on the incidence of these diseases in the human population of SA. This study was initiated to establish baseline data so that further studies can be undertaken for such high-risk populations.

Brucellosis and coxiellosis are highly contagious zoonotic infections of humans and domestic animals [7, 8]. In SA, *Brucella abortus* and *B. melitensis* mainly infect cattle and small ruminants (sheep and goats), respectively, and also infect humans while brucellosis has not been reported in pigs [9, 10]. These species have been reported in SA, first in small ruminants and later in cattle as well as humans since the turn of the century [11–13], that later resulted in bovine brucellosis scheme to control brucellosis. The scheme focuses on controlling *B. abortus* by vaccination of S19 and serological testing followed by seropositive slaughter in high-risk bovines such as dairy and export with voluntary participation for other livestock [14]. WOAH recommends “unequivocal diagnosis of Brucella infections can be made only by the isolation and identification of *Brucella*,” but in situations where bacteriological examination is not practicable, diagnosis must be based on molecular or immunological methods [10]. WOAH recommends serological tests for the control of brucellosis at the national or local level such as Rose Bengal test (RBT), indirect ELISA (iELISA), complement fixation test (CFT), and fluorescent polarization assay (FPA) as suitable screening tests [10]. The diagnostic performance characteristics of ELISAs and FPA are comparable with or better than that of the CFT, and as they are technically simpler to perform and more robust, their use may be preferred [10]. The problem with SA partial brucellosis scheme focusing only on bovines is once the disease has been established in a herd, it is difficult to control as it has variable incubation periods ranging from several months to at least two years and even nine years as reported [15]. These problems make control and elimination from herds costly and difficult as it takes a minimum of two years after removal of infected animals to declare a herd free and decades to declare countries free of brucellosis [15]. As human brucellosis is mainly caused by contact with *Brucella*-infected animals that could be asymptomatic [16], their secretions and carcasses result in occupational public health risks for those workers in contact with animals such as abattoir workers, animal handlers, and veterinarians. Despite the endemic occurrence of brucellosis in SA, only a few reports in humans are available [17].

Coxiellosis in animals is caused by *C. burnetii*, which is a leading cause of abortions, decreased reproductive ability, and subclinical infections in ruminants [18]. *Coxiella burnetii* has a biphasic development cycle. The large-cell variant is a vegetative form found in infected cells, while the small-cell variant is the extracellular infectious form shed in milk, urine, vaginal secretions, semen, and faeces in both animals and humans. The latter infectious form is also found in high concentrations in placental tissue and amniotic fluid [19], that persists for weeks to years in the environment [19]. Similar to brucellosis, humans are infected with coxiellosis through infected animals which may be asymptomatic [20, 21]. Their secretions result in occupational public health exposure potential for humans in contact with animals such as abattoir workers, animal handlers, and veterinarians. In SA, the first human case of Q-fever was documented in 1950 [22] and *C. burnetii* has been reported in goats, cattle, and sheep [23, 24] despite lack of surveillance programs.

*Toxoplasma gondii* is a ubiquitous protozoan parasite of warm-blooded animals [25], causing infections in humans and wild and domestic animals, including birds, cats, sheep, goats, cattle, and pigs [26]. Despite the organism infecting humans, wildlife, and domestic animals, only members of the cat family (Felidae) have been confirmed as definitive hosts for *T. gondii* infection. Unlike in humans, the clinical signs of toxoplasmosis infections in animals are nonspecific [27, 28], making diagnosis in animals by observation of symptoms insufficient. The molecular and serological tests such as PCR, indirect hemagglutination assay, indirect fluorescent antibody assay, latex agglutination test, and ELISA tests are currently used [29]. Serological results must be evaluated carefully because, according to bioassay investigations, most seropositive cattle lacked signs of active *T. gondii* infections [30]. Despite the infection in cattle, abortions, and neonatal mortality are not common [31], in contrast, infection with *T. gondii* significantly contributes to abortion and stillbirth in sheep, goats, cervids, and pigs [32, 33]. Toxoplasmosis is an under-reported parasitic infection in Africa [34]. There are limited studies on toxoplasmosis in SA; however, seropositive animals (cattle, sheep, and cats) have been reported [35].

The development of persistent zoonotic transmission from initial spillover incidents requires the interaction of complex mechanisms [36]. Conversely, there is general agreement that interaction between people and animals and their bodily fluids, whether directly or indirectly, is necessary for a successful interspecies transmission [36]. In developing countries, 25% of the infectious disease burden is due to zoonotic diseases [37], as poverty raises the risk of zoonotic diseases spreading in communities where people are in close contact with livestock and wildlife [38, 39]. The World Health Organization (WHO) reported an estimation of 600 million cases due to food-borne infections in 2010,while pathogenic bacteria were responsible for 350 million deaths of these cases [40]. Livestock and wildlife play a major role in the economies of many developing countries by providing food, income, and employment for the communities [41]. In low-income communities, livestock also serve as a source of wealth, means of transportation, and organic fertilizer for production [41]. Therefore, the burden of infections in livestock may result in reduced productivity and reduction in the number of live animals due to increased death rates, abortions, and reduced meat and milk production [41].

Globally, there is a high demand on food due to the increasing human population [42]. The implication is that there will be high demand for animal protein. This means that more animals will be slaughtered to meet the demand. Abattoir surveillance of diseases in slaughtered animals using serological and cultural assays may be used to diagnose newly introduced diseases and monitor disease control and eradication efforts. Information generated from abattoir surveillance could provide an early warning system for impending epidemics or failures of intervention programs such as livestock vaccination against certain diseases, thereby allowing early intervention efforts to prevent epidemic loss of animals and infections in humans. The usefulness of data obtained from abattoirs during surveillance for selected diseases is however dependent on the accuracy of the data obtained [43, 44]. This study focused on investigating the presence of zoonotic diseases that are mainly asymptomatic except for causing abortion, namely, brucellosis, coxiellosis, and toxoplasmosis in slaughtered livestock at abattoirs in the Eastern Cape Province. The objective was to determine the seropositivity of infectious zoonotic pathogens and the coexposure of the abovementioned diseases in slaughtered animals.

## 2. Materials and Methods

### 2.1. Study Area

**Figure 1:**
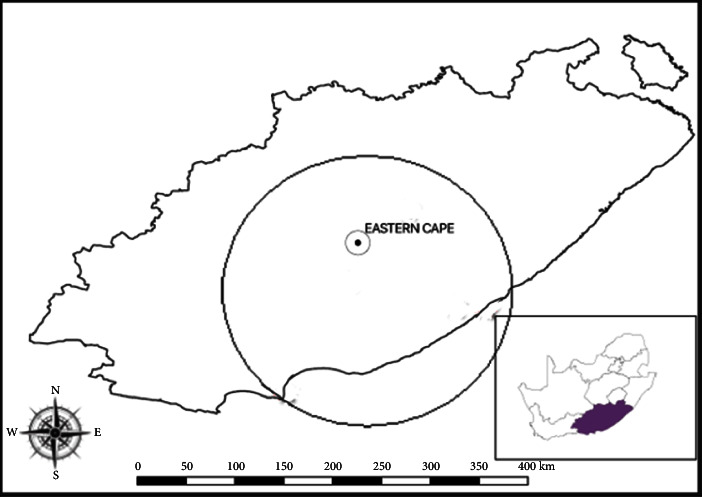
The Eastern Cape Province in South Africa is highlighted in purple in the South African map and selected abattoirs located within the circle in the Eastern Cape Province were sampled.

A cross-sectional study was conducted to investigate the seropositivity of brucellosis, coxiellosis, and toxoplasmosis and to report coexposures in livestock (cattle, sheep, and pigs) slaughtered at five abattoirs in the Eastern Cape Province (ECP) of South Africa (Figure 1). These abattoirs received livestock from ECP and neighbouring provinces (Free State and Kwazulu-Natal) except Western Cape Province, which is the only province in the country implementing movement control. ECP is the second largest province in the country (169,966 km^2^), with a human population of more than six million [45]. This area is surrounded by twelve registered abattoirs, including both high and low throughput abattoirs.

### 2.2. Selected Abattoirs

In this study, seven abattoirs were randomly selected in the study area (Figure 1); however, only five consented to participate. These comprised of high throughput (*n* = 3), slaughtering more than 20 livestock per species per day, and low throughput abattoirs (*n* = 2) slaughtering less than 20 livestock per species per day. The abattoirs are located more than 100 km apart from each other, with the exception of one low throughput abattoir, being in close proximity to one of the high throughput facilities. The managers of the selected abattoirs were notified about the research project and their consent to participate in the study was obtained before the study commenced.

### 2.3. Sampling and Data Collection

A randomly chosen subset of a single species was used to sample animals in a sequential manner. A total of 565 serum samples (280 cattle, 200 sheep, and 85 pigs) were randomly collected from five abattoirs between 2020 and 2022. Therefore, a total of 160, 115, and 290 samples were collected in 2020, 2021, and 2022, respectively. In addition to serum sampling, tissues (liver, spleen, kidney, lymph nodes, and tonsils) were collected from corresponding carcasses. Tissue samples from seropositive animals were cultured for *Brucella* isolation. Isolated *Brucella* species were confirmed with PCR. Approximately 50 ml of unclotted blood was collected at the point of slaughter from the jugular vein into sterilized cups, with 5 ml of the blood aliquoted into yellow-capped vacutainer tubes. Information regarding the animals was obtained from the abattoir managers, which included the age, sex, breed, and the origin of the animals. However, in some abattoirs, the animal origins were not provided. The blood samples were stored at 4–8°C prior to serum separation. The serum was aliquoted into 2 ml sample tubes, packaged in accordance with the National Road Traffic Act, 1996 (Act No. 93 of 1996), and transported to the Department of Veterinary Tropical Diseases, University of Pretoria, South Africa.

### 2.4. Sample Size and Data Analysis

The sample size was calculated using the following formula: *n* = *z*^2^*P*_exp_*Q*/*L*2, where *n* is the sample size, *P*_exp_ is the expected prevalence, *L* is the precision of the estimate (also called “the allowable error” or margin of error) which is equal to half the width of the confidence interval, *Q* = 1 − *P*_exp_, and *Z* is the (1 − *α*^2^) percentile of a standard normal distribution [45]; for *α* = 0.05, *Z* = 1.96. Due to lack of recent data on seropositivity of *Brucella* spp., *T. gondii*, and *C. burnetii* in cattle, sheep, and pigs in Eastern Cape Province, 11% prevalence was used based on recent data from other provinces in South Africa to determine the sample size [11, 35]. This provided the sample size of 151 for each species which meets the required sample size per the calculation, except for the porcine samples as fewer pig animals were received by the abattoirs during the sampling period. Data were cleaned and managed in Microsoft Excel® spreadsheet and the descriptive analysis (reporting the seropositivity, 95% confidence interval, and their associated *P* values) was performed using Stata V 14 (StataCorp, College Station, TX, USA).

### 2.5. Serological Testing

The criteria for *Brucella* seropositivity require the results of two or more of the techniques listed below for the valid confirmation of a sample.

#### 2.5.1. Brucellosis Rose Bengal Test (RBT)

The RBT was conducted using antigens and controls provided by Onderstepoort Biological Products (OBP). The testing was conducted according to the World Organization for Animal Health (WOAH) protocol [46]. Based on past validation tests, it has been determined that the RBT's diagnostic sensitivity and specificity are 100.00% and 75.00%, respectively [47, 48]. Briefly, serum samples were removed from the freezer to the fridge to defrost overnight. The samples were kept at room temperature prior to testing. A volume of 50 *µ*l of each serum sample was added into each well, and the formation of bubbles was avoided. An equivalent volume of 50 *µ*l of a positive control and a negative control were dispensed into separate wells as per the protocol. An equal volume of the anti-*Brucella* antigen was dispensed into each well containing the sera and positive and negative controls. The plate was agitated on a rocker for 4 minutes. Samples were considered positive for RBT when a visible agglutination was observed. The mean sensitivity and specificity were 81.2% and 86.3% reported by Gall and Nielsen [49] when most tests were not credited for ISI 17025 and 9001, while Chisi et al. [50] reported diagnostic sensitivity and specificity in SA bovine to be 95.8% and 100%.

#### 2.5.2. Brucellosis Complement Fixation Test (CFT)

The CFT was conducted at a South African National Accreditation System (SANAS) approved Serology laboratory at Agricultural Research Council-Onderstepoort Veterinary Research (ARC-OVR) in South Africa according to the WOAH protocol. The cut-off value for this test was ≥30 IU/ml as an indicator of infection. The mean sensitivity and specificity were 89.0% and 83.5% reported in [49], when most tests were not credited for ISI 17025 and 9001, while Chisi et al. [50] reported diagnostic sensitivity and specificity in SA bovine to be 93.9% and 100%.

#### 2.5.3. Brucellosis and *Coxiella burnetii* Enzyme-Linked Immunosorbent Assay (ELISA)

The serological tests to detect the presence of antibodies against *Brucella* spp. and *C. burnetii* were confirmed using indirect enzyme-linked immunosorbent assay (iELISA). Each sample was assessed in duplicate to reduce variance. The *Brucella* spp. and *C. burnetii* iELISA were conducted using the ID Screen brucellosis serum indirect multispecies (France) and ID Screen Q-Fever indirect multispecies kits (France), respectively. Both assays were performed as per manufacturer's instructions and the optical densities were read using the BioTek ELISA reader. Briefly, the frozen serum samples were stored at 4°C overnight to defrost and brought to room temperature prior to testing. The samples were vortexed and 10 *µ*l of the samples were transferred into the *C. burnetii*antigen-coated plates and the purified *Brucella* LPS (lipo-polyssacharide)-coated plates, respectively. A positive and negative control supplied with each kit was added into the plate wells, and the plate was incubated at 25°C for 45 min. The plate was then washed three times with 300 *µ*l of the wash solution (1X) using Bio-Rad PW40 microplate washer. The conjugate buffer (100 *µ*l) was then transferred into the washed plate and incubated at 25°C for 30 minutes. The above wash technique was repeated and 100 *µ*l of the substrate solution was added into each well. The plates were covered with foil and incubated in the dark for 15 minutes. Following the incubation, 100 *µ*l of the stop solution was transferred into each well and the plates were read at 450 nm. The results were interpreted as specified by manufacturer's instructions. The mean brucellosis iELISA sensitivity and specificity were reported by 96.0% and 93.8% by [49], when most tests were not credited for ISI 17025 and 9001, while Chisi et al. [50] reported diagnostic sensitivity and specificity in SA bovine to be 95.8% and 92.5%.

#### 2.5.4. *Toxoplasma gondii* Mast® Toxoreagent Test

A commercial latex agglutination test (LAT) produced by the Eiken Chemical Company of Japan, supplied by Davies Diagnostics (Pty.) Ltd., was used for detection of IgM and IgG antibodies against *T. gondii* and the reactions were performed using the U-shaped bottom microplates. The samples were processed at dilutions of 1 : 16 to 1 : 2048. An agglutination titer of 1 : 64 dilution was considered as a positive cut-off level for *T. gondii* antibodies [34, 51].

### 2.6. Isolation of *Brucella* spp. from Seropositive Livestock

The seropositive samples were processed according to set laboratory protocols in a bio‐safety laboratory level (BSL) 2^+^. About 200 *μ*l homogenates of the tissues from seropositive animals (17/565) (liver, kidney, spleen, and lymph nodes) were inoculated onto the modified CITA media [52] and incubated at 37°C with 5.0% CO_2_ for 5–14 days. Culture plates were considered negative and discarded following 14 days of incubation with no growth observed. The mean sensitivity and specificity were reported as 46.1% and 100%, respectively [49].

### 2.7. Genomic DNA Extraction from *Brucella* Isolates

DNA was extracted from the suspected isolates for *Brucella* spp. screening. This was done using the Pure-Link Genomic DNA kit according to the instructions of the manufacturer.

### 2.8. *Brucella*-Specific PCR and *Brucella* Species Confirmation

DNA amplification for detection of *Brucella* region using genus-specific 16S-23S rRNA interspacer region (ITS) was used for the detection of *Brucella* spp. [53]. Briefly, a PCR master mix of 12 *μ*l was prepared as follows: 6.5 *μ*l DreamTaq polymerase, 0.3 *μ*l (0.2 *μ*M) forward primer, 0.3 *μ*l reverse primer (0.2 *μ*M), and 4.9 *μ*l of nuclease-free water (Thermo Fisher Scientific, South Africa). From each sample, 3 *μ*l of DNA was used in a 15 *μ*l PCR reaction. The mix was amplified on a thermal cycler (Veriti 96 well) with a heated lid, preheated to 105°C. The PCR cycling condition consisted of 95°C for 3 minutes, followed by 35 cycles of 95°C for 1 minute, 60°C for 2 minutes, 72°C for 2 minutes, and a final extension of 72°C for 5 minutes. The target DNA region has a product size of 214 bp.

Multiplex AMOS-PCR which identified and differentiated *B. abortus* (F-GAC GAA CGG AAT TTT TCC AAT CCC), *B. melitensis* (F-AAA TCG CGT CCT TGC TGG TCT GA), *B. ovis* (F-CGG GTT CTG GCA CCA TCG TCG GG), *B. suis* (F-GCG CGG TTT TCT GAA GGT GGT TCA), and IS711 (R-TGC CGA TCA CTT AAG GGC CTT CAT) was conducted as described [53]. The four species-specific forward primers were used at a final concentration of 0.1 *μ*M with 0.2 *μ*M reverse primer IS711. PCR cycling condition consisted of an initial denaturation at 95°C for 5 minutes followed by 35 cycles of 95°C for 1 minute, 55.5°C for 2 minutes, 72°C for 2 minutes, and a final extension step at 72°C for 10 minutes. Multiplex Bruce-ladder PCR was conducted as described [53] to differentiate virulent *Brucella* spp. from S19 vaccine strain.

## 3. Results

A total of 565 sera samples were analysed with 49.56% cattle, 35.40% sheep, and 15.09% pigs. The location of some animals was unknown, but it was established that the animals were transported from other provinces except for Western Cape Province, which requires an animal movement permit. Of all the 565 samples collected, 276 (48.85%) were females while 289 (51.15%) were males (Table 1). Within the 280 cattle samples, 162 (57.86%) were females which included 3 heifers and 118 (42.14%) were males which included 15 males less than 2 years old (Table 2).

### 3.1. Brucellosis Seropositivity

No statistical association was found between *Brucella* seropositivity with species and sex on iELISA and RBT, while breed on iELISA was not a statistically significant factor. However, we found an association with age on both tests, and with breed only on RBT (Table 1). Of the 565 animals sampled, 4 (0.71%, 95% Cl: 0.26–1.87) were positive on iELISA and 15 (2.65%, 95% Cl: 1.6–4.36) were positive on RBT. Among the 280 cattle tested, 3.57% (10/280) were positive on RBT, 1.42% (4/280) were positive on iELISA while only one of the 10 (10%) RBT positive sera was confirmed positive on CFT, 0.36% (1/280). Of the 4 iELISA positive cattle, only 2 were positive on RBT. Antibodies against *Brucella* were detected in sheep 2% (4/200) and pigs 1.18% (1/85) using RBT. No antibodies were detected against *Brucella* in sheep and pigs using iELISA (Table 1). Details of seropositivity segregated by sex and age of each animal species are outlined in the Supplementary Tables 1 and 4, respectively.

### 3.2. Brucella DNA, Species Identified from Cultures, and Positivity

**Figure 2:**
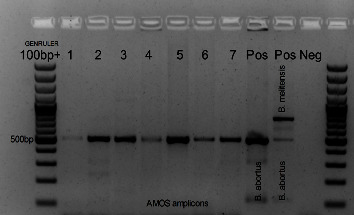
The 2% agarose gel imaging demonstrating positive results by conventional PCR amplicons of 498 bp *Brucella abortus* target and 730 bp *B. melitensis* target. Lanes 1–7 are *Brucella* isolates from tissue samples, lane 8 (POS) is *B*. abortus, lane 9 (POS) is a mixed control of *B. abortus* and *B. melitensis* positive controls, and lane 10 is the negative control.

No pure *Brucella* cultures were obtained as slow growing *Brucella* colonies were overgrown by faster growing contaminant and thus PCR assays were used to identify *Brucella* and the species on isolates (Figure 2). Most Brucella-like colonies were subcultured two to three times and each time the colonies were tested with *Brucella*-specific ITS PCR and then followed by AMOS-PCR assay but remained impure.

*Brucella*-specific DNA was detected in 11 (64.71%) of the 17 seropositive animals using the AMOS-PCR assay. Of the 10 RBT seropositive cattle, 7/10 *Brucella*-specific DNA was detected and identified as *B. abortus* (Figure 2). Of the 4 iELISA seropositive cattle, 4/4 *Brucella*-specific DNA was detected and identified as *B. abortus.* Thus, of the 12 RBT or iELISA seropositive cattle, 3.57% (10/280) were seropositive and PCR positive for brucellosis. Bruce-ladder PCR was used to differentiate between the field and vaccine strain, of which it was verified that there was no S19 vaccine strain isolated. Of the 4 seropositive sheep (RBT), 2/4 animals were culture positive for *Brucella*. *Brucella*-specific DNA was detected and identified as *B. melitensis* and *B. abortus* mixed infection for 1 animal while a *B. abortus* isolate was obtained from the second sheep. Thus, 1% (2/200) of the sheep was serology and PCR positive for brucellosis. There was no *Brucella* isolation from the seropositive pig sample. *Brucella* positivity of samples from Eastern Cape abattoirs were 4.3% (12/280) cattle, 1.0% (2/200)sheep, and 0/85 pigs based on at least two tests namely RBT, iELISA, or AMOS-PCR.

### 3.3. *Coxiella burnetii* Seropositivity

Seropositivity rates differed significantly between species for *C. burnetii* (*P* < 0.001). Of the 565 animals sampled, 106 (18.76%, 95% Cl: 15.74–22.20) were positive for *C. burnetii* antibodies. The highest percentage was observed in cattle 26.43% (74/280), followed by sheep 15% (30/200), and the lowest was pigs 2.35% (2/85). Exposure levels for *C. burnetii* also differed significantly by sex (*P* < 0.001). The highest frequency of *C. burnetii* antibodies was observed in females 25.72% (71/276) compared to males, 12.11% (35/289). A significant difference was further observed by age (*P* < 0.001). Livestock older than 2 years but less than 3 years had the highest positivity rate of 38.60% (22/57), followed by those older than 3 years at 17.10% (79/462), and the lowest was those between 1 and 2 years, 10.87% (5/46) (Table 3). Details of seropositivity segregated by sex and age of each animal species are outlined in the Supplementary Tables 2 and 3, respectively.

### 3.4. *Toxoplasma gondii* Seropositivity

The seropositivity differed significantly between species, sex, and age for *T. gondii* (*P* < 0.001). Of the 565 animals sampled, 115 (20.35%, 95% Cl: 17.22–23.89) were positive for *T. gondii*. A relatively increased proportion of seropositivity was observed among cattle 37.86% (106/280), pigs 7.06% (6/85), and sheep 1.50% (3/200). Antibodies against *T. gondii* were detected more in females, 31.16% (86/276), than in males, 10.03% (29/289). Livestock older than 2 years but less than 3 years had the highest positivity rate of 33.33% (19/57), followed by those older than 3 years, 19.05% (88/462), and the lowest was those between 1 and 2 years, 17.39% (8/46) (Table 3). Details of seropositivity segregated by sex and age of each animal species are outlined in Supplementary Tables 2 and 3, respectively.

### 3.5. *Brucella*, *C. burnetii*, and *Toxoplasma gondii* Coexposure in Livestock Species

**Figure 3:**
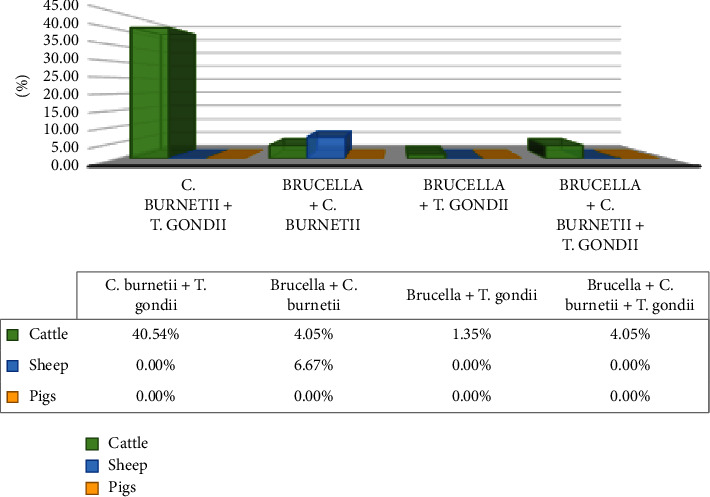
Seropositivity profile of zoonotic infection coexposure in livestock species.

Evidence of antibody coexposure against *Brucella*, *C. burnetii*, and *T. gondii* was observed in cattle. However, there was no coexposure observed in pigs. Coexposure for *C. burnetii* and *T. gondii* 40.54% (30/74) was detected from cattle. The proportion of cattle with antibodies against *Brucella* and *T. gondii* was observed at 1.35% (1/74), while the coexposure against Brucella and C. *burnetii* 4.05% (3/74). Cattle serum samples obtained from two different abattoirs had coexposure against *Brucella*, *C. burnetii*, and *T. gondii* 4.05% (3/74). Sheep samples had a coexposure against *Brucella* and *C. burnetii* 6.67% (2/30) (Figure 3).

## 4. Discussion

This study investigated the presence of zoonotic infections causing abortions in livestock which included *Brucella*, *C. burnetii*, and *T. gondii* exposure, in apparently healthy livestock (cattle, sheep, and pigs). Despite the serious public health threat and economic impact these diseases have on livestock in South Africa, there is no surveillance program for these pathogens except for the partial bovine brucellosis scheme that focusses on high-risk bovines. Thus, abattoirs present an ideal passive surveillance opportunity for zoonotic infections in livestock. However, in a previous study, the trace-back of animals to a farm was limited especially with animals from feedlots and communal areas [11]. We also investigated the coexposure and discussed the significance of these pathogen diagnostics in the relevant livestock species and possible health risks to humans handling these animals, such as abattoir workers and animal handlers. This study demonstrated the presence of antibody/antigen coexposure against (i) *Brucella*, *C. burnetii*, and *T. gondii* in cattle, (ii) *Brucella* and *T. gondii* in sheep and cattle, and (iii) no coexposure observed in pigs.

Coexposure of *Brucella*, *T. gondii*, and *C. burnetii* in cattle has not been reported in the literature. Although more than one-third of the serum samples were *T. gondii* positive in cattle, this result should be considered with caution due to the frequent lack of evidence of viable *T. gondii* infections in seropositive cattle. There are very few reports on naturally exposed cattle with positive *T. gondii* bioassays indicating active infections [54, 55]. Another South African study has reported codetection of *C. burnetii* and *T. gondii* (15.2%) in cattle from a communal farming area at the wildlife-livestock-human interface [56]. Despite toxoplasmosis not commonly leading to reproductive pathologies in cattle, the organism results in the formation of cyst in the tissues posing a risk to public health if the contaminated meat is consumed undercooked [57]. Also, in a recent publication from India, a 2.21% coexposure of *C. burnetii* and *T. gondii* was documented in dairy cattle, resulting in reproductive disorders [58]. Coexposures of *Brucella* and *C. burnetii* in cattle and sheep are significant as they result in reproductive losses, and both have public health issues for humans in contact with infected animals [59, 60].

This study observed an overall *Brucella *positivity of 4.3% and 1.0% in cattle and sheep, respectively. A study conducted from 2007 to 2015 had increased seropositivity by 6.31% in cattle, followed by 2.09% in sheep and 0.63% in pigs [61]. Our study has reported 17 *Brucella* positive animals using all three serological tests, of which 66.7% were females. This high seropositivity rate in females poses zoonotic risks knowing the transmission route of the infection from animals to humans includes ingestion of contaminated milk [62]. This finding agrees with other studies which found definitive diagnosis difficult in males [63, 64]. The reason is that females are kept in the breeding herd for a substantially longer period than males, increasing their risk of infection [65]. Although we are not privy to the exact animal origin details, there are 23 possible herds (farms) sampled between 2020 and 2022 at the abattoirs according to slaughter records. The cattle made up 14 potential herds, while the sheep and pigs potentially made up 5 and 4 farms, respectively. Brucellosis is a herd disease so it is safe to assume that animals from the same herd would test positive. The low seropositivity in cattle could, therefore, be due to the sample distribution according to origin. Of the 14 potential cattle herds tested, the 17 positive animals were distributed within 6 herds. The 6 herds were predominantly from 2 provinces.

Although our seropositivity was low, our study has shown similar technique results to a study conducted by Kolo et al. [11] in Gauteng Province (SA) where they reported 11.0% RBT, 5.5% iELISA, and 2.0% CFT in cattle. Our study reported increased *Brucella* seropositivity of RBT (3.57%), followed by iELISA (1.42%) and CFT (0.36%). Our study had higher seropositivity in detecting antibodies against *Brucella* with the use of RBT compared to iELISA and CFT. The RBT is considered an exceptionally sensitive method to detect *Brucella*-specific IgG1, IgM, and potentially IgG2 antibodies in cattle serum [66]. The RBT is primarily used as a standard test due to the high sensitivity of the assay, which might result in false-positive results [67]. Although a previous South African study demonstrated no statistically significant difference in these tests' ability to diagnose *Brucella*, the RBT and ELISA showed an increased sensitivity and specificity when combined [68]. Also, these tests are validated on symptomatic animals or animals with documented pathology. The CFT is highly specific but may result in false negatives due to its low sensitivity [68], especially in asymptomatic animals. Our study further shows that the *Brucella* seropositivity is significantly higher in younger animals as compared to older animals. One would assume this could be due to latent infections in heifers being born from infected cows or due to vaccination with S19 as the study has shown increased positivity in females. However, there were only 3 heifers (<2 years) in our study. The remaining positives were from young males (<2 years), which precludes vaccination since only females are vaccinated, thus suggesting a higher exposure of the pathogen in young cattle. Previous studies have reported an increased seropositivity in older animals than in younger animals Segwagwe et al. [67, 69]. We also observed higher positive cases among Bonsmara as compared to the Holstein breeds in cattle. Dairy herds consisting of Holsteins are farmed intensively, where reproductive issues are readily diagnosed and well documented [70]. The AMOS-PCR identified *B. abortus* in cattle and a mixed infection of *B. abortus* and *B. melitensis* in sheep (64.71%) of the seropositive samples.

In this study, *C. burnetii* seropositivity of 26.43%, 15.00%, and 2.35% was observed in cattle, sheep, and pigs, respectively. A similar study conducted in Gauteng Province (SA) from slaughtered livestock at red meat abattoirs had comparable results with the present study where they reported a high seroprevalence of *C. burnetii* in cattle 9.40%, followed by sheep 4.30% and the least in pigs 0.90% [71]. The seropositive cattle were reported to have originated from eleven potential herds, while the sheep sampled potentially came from three and the pigs were potentially from two farms. Due to the infectious nature of *C. burnetii*, comingling of animals would lead to a higher seropositivity. Historically, there have been serological crossreactions for *C. burnetii* [72], which may result in false positives leading to an increased seropositivity. There are also cross reactions of *C. burnetii* with *Coxiella*-like endosymbionts (CLEs), making definitive diagnosis difficult [73]; thus, the results are similarly interpreted with caution. We detected antibodies against *C. burnetii* in 25.72% (71/276) females and 12.11% (35/289) in males. Previous research has demonstrated the presence of *C. burnetii* in semen [74], consequently suggesting that the higher seropositivity in females may be due to multiple females being involved with a single male during reproduction management [71].

Age was a statistically significant factor in our study considering that exposure to *C. burnetii* was higher among animals >2-3 years (38.60%) and >3 years (17.10%). These findings may be due to increased possibilities of *C. burnetii* exposure with age. The unique spore-like structures that *C. burnetii* produces are exceptionally resistant to environmental factors [75, 76]. These traits allow the bacterium to survive in the environment for up to a year, thus increasing the potential for exposure to livestock across seasons. In this study, cattle breed differences were observed, with the highest seropositivity detected in cross-breed (35.71%), followed by Bonsmara (33.33%) and the least in Jersey (18.42%). The Dorper breed (33.33%) had a higher seropositivity as compared to the Merino sheep breed (11.76%).

The reported prevalence of *T. gondii* antibodies in livestock has varied greatly in Africa. In western African countries, seropositivity of *T. gondii* antibodies was 16.30%, 29.10%, and 35.90% in cattle, sheep, and pigs, respectively [77]. In southern African countries (Zimbabwe and South Africa), a prevalence in cattle of 20.00% (95% Cl: 5–39), pigs 13% (95% Cl: 1–29), and small ruminants (goats and sheep) 11.00% (95% Cl: 0–31%) was observed [78]. The findings of our study revealed 37.86% (106/280) seropositivity in cattle followed by pigs at 7.06% (6/85), and the lowest in sheep 1.50% (3/200). In livestock, chronic *T. gondii* infections have been extensively documented in pigs and small ruminants (sheep and goats), resulting in significant economic losses through abortions and birth of deceased or disabled progeny [79, 80]. According to studies, consuming raw or undercooked beef and milk increases the risk of contracting zoonotic infections including *T. gondii* [81]. It was reported in South Africa by Agricultural Research Council-Onderstepoort Veterinary Research (ARC-OVI) that, between 2007 and 2017, only 567 animal samples had been submitted for toxoplasmosis tests, further indicating the negligence of this zoonotic disease surveillance [35].

The latex agglutination test (LAT) used for *T. gondii* is preferably used as a screening test. In comparison to the indirect immunological fluorescence test, the LAT assay has a comparable higher sensitivity [34]. The presence of IgM antibodies in serum are detectable about 1 week following infection and may persist for months or years [29]. IgG antibodies indicate the existence of infection but give little information regarding when the infection first occurred [29]. We observed an increased *T. gondii* seropositivity in livestock between 2 and 3 years, 33.33% (19/57), followed by a decrease in animals older than 3 years, 19.05% (88/462). It should be noted that the sample numbers for livestock older than 3 years were larger than the preceding age group. This trend is contrary with studies reported in SA on sheep and West African cattle, sheep, and goat where the seroprevalence increased with the age of animals [34, 77]. Older animals, as opposed to young ones, are more likely to have a recurrence of *T. gondii* infection [82]. According to other studies, older animals may be more susceptible to infection due to environmental exposure of infective oocysts that they ingest or inhale [83]. By consuming uncooked meat infected with *Toxoplasma* cysts, scavenging cats around abattoirs can contract the infection and subsequently serve as sources of infection [83]. This will increase the risk of infection among humans and livestock surrounding the abattoirs.

## 5. Conclusion

This study demonstrated the exposure to *Brucella*, *C. burnetii*, and *T. gondii* in apparently healthy cattle, sheep, and pigs where some animals showed coexposure to the pathogens. The control of these selected zoonotic infections through vaccination programs is currently only possible for brucellosis; thus, the impact on public health is unknown. Furthermore, brucellosis detection is complicated by latency and limitation of serology tests and culture to detect chronic infected animals. Tests such as RBT are highly sensitive, but results can be confounded by the lower specificity and false positives. While ELISA is highly specific, the recommended cut offs can lead to false negatives. These asymptomatic diseases pose a threat to humans who handle animals, such as abattoir workers, veterinarians, and animal technicians. The detection of these pathogens largely depends on serological tests which are complicated by the risk of cross reaction to close-related species. Awareness regarding zoonotic infections is essential to sensitize laymen working in such industries to cultivate safe working practices and should be rolled out as part of control strategies.

## Figures and Tables

**Table 1:** Serological results of *Brucella* spp. in livestock slaughtered from abattoirs in Eastern Cape Province.

Variable	Category	No. of animals sampled	RBT			ELISA		
			Positive	% seropositive	*P* value	Positive	% seropositive	*P* value
Species	Cattle	280	10	3.57	0.212	4	1.43	0.211
	Pig	85	1	1.18		0	0.00	
	Sheep	200	4	2		0	0.00	

Sex	Female	276	10	3.62	0.154	10	3.62	0.056
	Male	289	5	1.73		5	1.73	

Age	1-2 years	46	3	6.52	0.005^*∗*^	0	0.00	0.006^*∗*^
	>2-3 years	57	5	8.77		3	5.26	
	>3 years	462	7	1.51		1	0.22	

Breed	Beef master	40	0	0.00	0.048^*∗*^	0	0.00	0.206
	Bonsmara	90	9	10		3	3.33	
	Cross-breed	14	0	0.00		0	0.00	
	Friesian	18	0	0.00		0	0.00	
	Holstein	80	1	1.25		1	1.25	
	Jersey	38	0	0.00		0	0.00	
	Dorper	30	0	0.00		0	0.00	
	Merino	170	4	2.35		0	0.00	
	Large white (pig)	85	1	1.18		0	0.00	

NB: statistical significance variables (<0.05) have been marked with a “^*∗*^”.

**Table 2:** Species results stratified by gender and age.

Species	Gender		Age (years)	No. of animals sampled
	Females	Males		
Cattle	162 (57.86%)	118 (42.14%)	1-2	18
			>2-3	38
			>3	224

Sheep	80 (40%)	120 (60%)	1-2	8
			>2-3	19
			>3	173

Pigs	34 (40%)	51 (60%)	1-2	20
			>2-3	0
			>3	65

**Table 3:** Serological evidence of *Coxiella burnetii* and *Toxoplasma gondii* in livestock slaughtered from abattoirs in Eastern Cape Province, SA.

Variable	Category	No. of animals sampled	*Coxiella burnetii*			*Toxoplasma gondii*		
			No. tested positive	% seropositive	*P* value	No. tested positive	% seropositive	*P* value
Species	Cattle	280	74	26.43	<0.001^*∗*^	106	37.86	<0.001^*∗*^
	Pig	85	2	2.35		6	7.06	
	Sheep	200	30	15		3	1.5	

Sex	Female	276	71	25.72	<0.001^*∗*^	86	31.16	<0.001^*∗*^
	Male	289	35	12.11		29	10.03	

Age	1-2 years	46	5	10.87	<0.001^*∗*^	8	17.39	<0.001^*∗*^
	>2-3 years	57	22	38.60		19	33.33	
	>3 years	462	79	17.10		88	19.05	

Breed	Beef master	40	10	25	<0.001^*∗*^	6	15	<0.001^*∗*^
	Bonsmara	90	30	33.33		43	47.78	
	Cross-breed	14	5	35.71		9	64.29	
	Friesian	18	4	22.22		14	77.77	
	Holstein	80	18	22.5		27	33.75	
	Jersey	38	7	18.42		7	18.42	
	Dorper	30	10	33.33		0		
	Merino	170	20	11.76		3	1.76	
	Large white (pig)	85	2	2.35		6	7.06	

NB: statistical significance variables (<0.05) have been marked with a “^*∗*^”.

## Data Availability

All the relevant data and supplementary information are included in the paper.

## Supplementary Materials

Supplementary Materials Supplementary information includes Brucella seropositivity results stratified by species and gender, *C. burnetii* and *T. gondii* seropositivity stratified by species and gender, *C. burnetii* and *T. gondii* seropositivity stratified by species and age, and Brucella seropositivity results stratified by species and age.
