# Supplementary material for: Serological Evidence and Coexposure of Selected Infections among Livestock Slaughtered at Eastern Cape Abattoirs in South Africa
Source: Int J Microbiol. 2023 Dec 1;2023:8906971. doi: 10.1155/2023/8906971 (PMC10708957; doi:10.1155/2023/8906971)
Supplement: Supplementary Materials — Supplementary information includes Brucella seropositivity results stratified by species and gender, C. burnetii and T. gondii seropositivity stratified by species and gender, C. burnetii and T. gondii seropositivity stratified by species and age, and Brucella seropositivity results stratified by species and age. [file 8906971.f1.docx]

**Supplementary data**

**Table 1: Brucella seropositivity results stratified by species and gender**

| Species | Gender | No of Animals sampled | RBT | | | iELISA | | | CFT | | |
| --- | --- | --- | --- | --- | --- | --- | --- | --- | --- | --- | --- |
|  |  |  | No tested positive | % | P-value | No tested positive | % | P-value | No tested positive | % | P-value |
| Cattle | Females | 162 | 7 | 4.32 | 0.428 | 4 | 2.47 | 0.086 | 1 | 0.62 | 0.393 |
|  | Males | 118 | 3 | 2.54 |  | 0 |  |  |  |  |  |
| Sheep | Females | 80 | 3 | 3.75 | 0.149 | 0 |  |  | 0 |  |  |
|  | Males | 120 | 1 | 0.83 |  | 0 |  |  | 0 |  |  |
| Pigs | Females | 34 | 0 |  | 0.338 | 0 |  |  | 0 |  |  |
|  | Males | 51 | 1 | 1.96 |  | 0 |  |  | 0 |  |  |

**Table 2: Coxiella burnetii and Toxoplasma gondii seropositivity results stratified by species and gender**

| Species | Gender | No of animals sampled | *Coxiella burnetii* iELISA | | | *Toxoplasma gondii* | | |
| --- | --- | --- | --- | --- | --- | --- | --- | --- |
|  |  |  | No tested positive | % | P-value | No tested positive | % | P-value |
| Cattle | Females | 162 | 58 | 35.80 | <0.001 | 81 | 50 | <0.001 |
|  | Males | 118 | 16 | 13.56 |  | 25 | 21.19 |  |
| Sheep | Females | 80 | 13 | 16.25 | 0.686 | 1 | 1.25 | 0.325 |
|  | Males | 120 | 17 | 14.17 |  | 2 | 1.67 |  |
| Pigs | Females | 34 | 0 |  | 0.243 | 4 | 11.76 | 0.167 |
|  | Males | 51 | 2 | 3.92 |  | 2 | 3.92 |  |

**Table 3: Coxiella burnetii and Toxoplasma gondii seropositivity results stratified by species and age**

| Species | Age | No of Animals sampled | *Coxiella burnetii* iELISA | | | *Toxoplasma gondii* | | |
| --- | --- | --- | --- | --- | --- | --- | --- | --- |
|  |  |  | No tested positive | % | P-value | No tested positive | % | P-value |
| Cattle | 1-2 | 18 | 4 | 22.22 | 0.143 | 8 | 44.44 | 0.328 |
|  | >2-3 | 38 | 15 | 39.47 |  | 18 | 47.37 |  |
|  | >3 | 224 | 55 | 24.55 |  | 80 | 35.71 |  |
| Sheep | 1-2 | 8 | 1 | 3.33 | 0.020 | 0 |  | 0.353 |
|  | >2-3 | 19 | 7 | 23.33 |  | 1 | 5.26 |  |
|  | >3 | 173 | 22 | 73.33 |  | 2 | 1.16 |  |
| Pig | 1-2 | 20 | 0 |  | 0.427 | 0 |  | 0.159 |
|  | >2-3 | 0 |  |  |  | 0 |  |  |
|  | >3 | 65 | 2 | 3.08 |  | 6 | 9.23 |  |

**Table 4: Brucella seropositivity results stratified by species and age**

| Species | Age | No of Animals sampled | RBT | | | iELISA | | | CFT | | |
| --- | --- | --- | --- | --- | --- | --- | --- | --- | --- | --- | --- |
|  |  |  | No tested positive | % | P-value | No tested positive | % | P-value | No tested positive | % | P-value |
| Cattle | 1-2 | 18 | 2 | 11.11 | 0.045 | 0 |  | 0.001 | 0 |  | 0.041 |
|  | >2-3 | 38 | 3 | 7.89 |  | 3 | 7.89 |  | 1 | 2.63 |  |
|  | >3 | 224 | 5 | 2.23 |  | 1 | 0.45 |  | 0 |  |  |
| Sheep | 1-2 | 8 | 0 |  | 0.020 | 0 |  |  | 0 |  |  |
|  | >2-3 | 19 | 2 | 10.53 |  | 0 |  |  | 0 |  |  |
|  | >3 | 173 | 2 | 1.16 |  | 0 |  |  | 0 |  |  |
| Pig | 1-2 | 20 | 1 | 5 | 0.167 | 0 |  |  | 0 |  |  |
|  | >2-3 | 0 |  |  |  | 0 |  |  | 0 |  |  |
|  | >3 | 65 | 0 |  |  | 0 |  |  | 0 |  |  |
